# A large-scale, LLM-assisted and validated dataset of biomass and waste conversion technologies and feedstocks

**DOI:** 10.1038/s41597-026-07820-0

**Published:** 2026-07-07

**Authors:** Zahir Barahmand, Lars-Andre Tokheim, Liang Wang, Morten Seljeskog, Marianne Eikeland

**Affiliations:** 1 University of South-Eastern Norway, Kjølnes Ring 56, 3918 Porsgrunn, Norway; 2 SINTEF Energy Research, Trondheim, Norway

## Abstract

Biomass, organic wastes and by-products are increasingly targeted for low-carbon fuels and value-added chemicals. However, strategic decision-making from a circular economy perspective requires a big-picture view of the relative significance of different conversion technologies in handling diverse feedstock portfolios, and no large-scale, cross-technology mapping of these portfolios is currently available. Thus, we assembled a literature-derived dataset linking eight major waste-to-X valorisation technologies (gasification, pyrolysis, hydrothermal liquefaction, torrefaction, anaerobic digestion, aerobic digestion, fermentation and transesterification) to their reported feedstocks. Using the Scopus database, 121,365 records were retrieved with harmonised search strings, spanning publications from 1887 to 2026, including a small number of early-indexed 2026 records. This constrained yet scalable search strategy facilitates automated extraction and validation while yielding a rich dataset. Further, an LLM-assisted workflow was implemented to extract candidate technology and feedstock phrases, followed by a two-level validation that combines rule-based cleaning with targeted LLM re-evaluation to minimise manual curation. The resulting dataset provides technology-specific, validated feedstock descriptors that support comparative analyses and decision-support applications in a circular bioeconomy context.

## Background & Summary

Limiting global warming to well below 2 °C^1^, and ideally 1.5 °C, can now only be reached if governments take concerted action on greenhouse gas emissions across all sectors^2^, and the IPCC Sixth Assessment Report (AR6)^3^ identifies bio-based energy carriers and carbon dioxide removal options as critical components of mitigation pathways while emphasising that sustainable biomass is a constrained resource that must be deployed where it delivers the highest system-level value. In this context, international and regional policy frameworks increasingly position biomass- and waste-derived fuels, chemicals and materials as central to decarbonising hard-to-abate sectors such as aviation, maritime transport and high-temperature industry^4–6^. In the European Union (EU), the European Green Deal^7^ and the EU Climate Law^8^ establish climate neutrality by 2050 and a binding target of at least 55% greenhouse gas reduction by 2030 relative to 1990 levels. The “Fit for 55” package^9^ operationalises these commitments across energy, transport, industry and land use, including dedicated mandates for renewable and low-carbon fuels in aviation (ReFuelEU Aviation^10^) and maritime transport (FuelEU Maritime^11^), and revises the Renewable Energy Directive^12^ accordingly. The recast Renewable Energy Directive (RED II; Directive (EU) 2018/2001) and its 2023 amendment (RED III)^13^ further set binding renewable energy targets and dedicated sub-targets for advanced biofuels produced from Annex IX feedstocks, with a strong emphasis on lignocellulosic residues, wastes and other non-food biomass. Collectively, these policy trajectories, reinforced by national bioeconomy and circular economy strategies, have accelerated research and deployment of biomass and waste conversion pathways that transform diverse biogenic residues, organic wastes and dedicated biomass into fuels, energy carriers and value-added chemicals.

The conversion landscape for biomass, organic wastes, and biogenic by-products can be organised into four broad families, thermochemical routes, hydrothermal routes, biological or biochemical routes, and physicochemical routes^14–19^. Thermochemical options include combustion or incineration, pyrolysis, gasification, and torrefaction^20–23^. Hydrothermal routes include hydrothermal liquefaction, hydrothermal gasification, hydrothermal oxidation, hydrothermal hydrolysis, and hydrothermal carbonisation, which are often well matched to wet feedstocks^24–28^. Biological and biochemical options include anaerobic digestion, composting, and fermentation, while enzymatic hydrolysis is commonly used as a saccharification step to enable downstream microbial conversion^29–34^. Physicochemical routes primarily cover catalytic upgrading of lipid-rich feedstocks through esterification and transesterification^35^. Integrated chains also appear in the literature, such as thermochemical syngas production followed by microbial conversion, which can be represented as connected unit processes rather than a separate top-level pathway^36,37^.

This rich conversion landscape defines a broad technology-selection space. Each technology pathway has a distinct sustainability profile, with different implications for resource use, emissions, co-products and system integration across the bioeconomy. Well-known examples, such as the suitability of hydrothermal routes for wet feedstocks^38,39^ or transesterification for lipid-rich feedstocks^40,41^, provide useful technology-specific knowledge, but they do not give a systematic, cross-technology view of the full feedstock–technology evidence base. This raises a decision-support question: for a given feedstock and context, which conversion route, or combination of routes, should be considered? The criteria can be framed from several perspectives, including climate mitigation, cost, energy security, technology readiness and social acceptance.

In this work, we adopt a strategic upstream circularity lens. By “upstream”, we mean a portfolio-level view of the feedstock side of the comparison, rather than a process-specific performance assessment. “Strategic” means a screening-level comparison intended to inform big-picture choices before more data-intensive analyses such as life cycle assessment (LCA), techno-economic analysis (TEA) or process simulation are undertaken. We use “circularity” in a bounded sense, following the CE-Core/CE-Plus^42^ distinction: feedstock-related value retention, recovery and loop closure form the circularity core, while ethical concerns, externalities, regulatory conditions and technological feasibility are treated as CE-Plus elements only when they directly enable, govern or evaluate circular resource use, rather than when they represent general sustainability concerns^43–46^.

Under this lens, the relevant question is not only whether a technology can process a given feedstock, but what kind of circular value that feedstock use represents. This depends on the feedstock’s material status, for example whether it is a primary or virgin resource, a market commodity, a non-hazardous residue or by-product, or a hazardous or potentially hazardous secondary stream. It also depends on conditions such as baseline fate, competing uses, and regulatory or hazard status. Circularity therefore cannot be inferred from renewable origin or technical convertibility alone^47–51^. For example, if a technology is reported with both primary biomass and hazardous or potentially hazardous secondary materials, the strategic question is not simply whether both can be converted, but which feedstock use carries stronger circularity interest and what additional evidence is needed on sourcing, composition, handling and regulatory conditions.

The dataset supports this lens by making explicit, for each technology, the range and type of feedstocks reported in the literature. It therefore provides the feedstock-side evidence needed to examine how far different technologies draw on and valorise secondary resource streams, and to combine this information with process-level analyses such as LCA, TEA or multi-criteria decision analysis (MCDA). The dataset can also support upstream extensions of portfolio-based decision tools such as the EcoStrategic Index^52^, which was developed to evaluate economic value creation through downstream product-portfolio diversity in waste-to-X technologies. In an upstream application, the same portfolio logic can be applied to feedstock descriptors by comparing the breadth, diversity and circularity relevance of the feedstock base associated with each conversion route.

To our knowledge, no prior study provides a harmonised, record-level feedstock–technology mapping across biomass- and waste-to-X technologies at this scale. Previous studies have related feedstocks to conversion technologies, but usually within a single technology or a limited set of technologies, and through literature syntheses with different scopes, selection criteria and classification schemes. Examples include Hameed *et al*.^53^ for gasification of municipal-solid-waste and biomass blends, Barahmand *et al*.^21^ for pyrolysis, Jha *et al*.^54^ across thermochemical conversion routes, and Zhu *et al*.^55^ across selected waste categories. These studies are valuable for understanding feedstock suitability within specific technological contexts, but their feedstock groupings cannot be directly compared across technologies because they were not produced using a common sampling, extraction and labelling protocol. A consistent cross-technology view therefore requires a large, harmonised and reproducible dataset in which the same methodological logic is applied to each technology.

The present dataset addresses this need. It is not intended to serve as an exhaustive bibliography for any single technology. Instead, it is a deliberately constrained and consistently sampled dataset defined by a harmonised “[technology] of” search pattern, such as “gasification of” or “pyrolysis of”, applied to title, abstract and keyword fields. This design trades exhaustive coverage for consistency, reproducibility and scalability; studies that describe a feedstock–technology relationship in different wording may therefore be missed. Because each technology is sampled using the same search logic and processed through the same extraction and classification workflow, feedstock–technology patterns can be compared across technologies without the selection differences that typically affect narrative or technology-specific reviews. The resulting record counts should therefore not be interpreted as comprehensive measures of research activity. Rather, they represent a structured and comparable evidence sample optimised for large-scale feedstock–technology mapping.

Eight major valorisation technology families were targeted, spanning the four main conversion routes: thermochemical, hydrothermal, biological/biochemical and physicochemical. Information was extracted from 121,365 scientific studies. The dataset is constructed using a multi-step, large language model (LLM) assisted pipeline for data extraction^56,57^, cleaning and validation from titles and abstracts, followed by structured rule-based processing and manual expert curation, thus combining the scale needed to characterise a broad, cross-technology decision space with the reliability required for circular-economy-oriented analyses. The resulting data structure links each bibliographic record to a validated primary feedstock descriptor and technology label, enabling consistent querying across technologies and feedstock classes. Comparable LLM-assisted extraction pipelines have recently been adopted in Scientific Data for constructing domain-specific corpora from scientific literature and for deriving structured spatiotemporal records from judicial text, where LLM outputs are systematically combined with rule-based post-processing and expert validation^56,58,59^.

The dataset is intended as a reusable resource for evidence mapping, sustainability assessment and circular-economy analysis. Detailed reuse scenarios and practical considerations for using the dataset are provided in the Usage Notes section.

## Methods

### Clarifying aims and objectives

The workflow is designed to ensure transparency, consistency and reproducibility, drawing on the principles of the PRISMA 2020 guidelines^60^ while adapting them to a large-scale, data-extraction–oriented study. The primary objective of this data descriptor is to map, at very large-scale, the decision space defined by different feedstock types across eight major biomass and waste conversion technologies, thus providing a basis for subsequent strategic decision-making studies. These technologies span the four conversion routes defined in this study: thermochemical (gasification, pyrolysis, torrefaction), biological (anaerobic digestion, aerobic digestion, fermentation), hydrothermal (hydrothermal liquefaction) and physicochemical (transesterification). These eight technologies were selected because they are among the most widely reported and feedstock-flexible conversion options in the biomass and waste literature, collectively covering all four conversion route families used in this study and enabling a consistent, scalable extraction protocol across technologies. Although combustion/incineration is historically a major biomass conversion route, it is primarily an energy-recovery pathway producing heat and/or power. The present dataset is intentionally scoped to conversion technologies that enable *waste-to-X* valorisation through storable intermediates and diverse product portfolios (e.g., fuels and platform chemicals), consistent with circular-bioeconomy decision-support use cases. In value-hierarchy frameworks such as the value pyramid of bio-products^52^, energy/heat occupies the lowest tier, and policy narratives likewise emphasize shifting from low-return bioelectricity and biofuels toward higher-value bio-based chemicals and materials. Therefore, combustion/incineration is treated as out of scope for the current dataset and may be addressed in a dedicated future extension.

In very large-scale studies, both data extraction and validation pose major methodological challenges. In our related works^61–64^, we have tested different configurations of purely manual extraction/validation and semi-automated, LLM-assisted extraction/validation (with full datasets and protocols provided in an open repository). Building on these lessons and given we expect tens of thousands of relevant records, we adopt a semi-automated, LLM-assisted strategy for both extraction and validation, structured around simple, transparent rules to support reproducibility and to reduce the burden of manual curation. We construct technology-specific search strings that not only retrieve a substantial and diverse corpus of studies but also impose a simple and interpretable structure on titles and abstracts. This pattern-based design provides explicit cues that support LLM-assisted extraction and validation and is expected to reduce systematic errors in the extraction phase.

Validation is organised as a multi-step procedure. Before formal validation, we apply preliminary data cleaning and harmonisation, not as a source of ground truth, but to structure the records in a way that facilitates more efficient and reliable validation. We use the same LLM family (the GPT-4o mini model accessed via the ChatGPT API, previously benchmarked in our other studies and found suitable for large-scale literature extraction) for both extraction and validation, but with distinct prompts and decision rules so that validation independently cross-checks, rather than simply reproduces, the extraction logic. If the number of mismatches between extraction and validation outputs remains within predefined thresholds, we perform targeted manual curation; if the mismatch rate is too high, additional rounds of automated validation and cleaning are executed before manual review.

For bibliographic retrieval, we use the Scopus database, primarily because its export functionality allows substantially larger batches than Web of Science (up to 20,000 records per export compared with 1,000), which is essential for very large-scale data extraction. Searches are performed on titles, abstracts and author keywords, without restrictions on publication year or language, to avoid temporal or linguistic bias and to capture the broadest possible evidence base for subsequent feedstock–technology mapping.

For coding, documentation and data handling, we structure the workflow such that each of the eight technologies is treated as a separate processing stream (with its own search, extraction, validation and intermediate files), which simplifies implementation and debugging, allows technology-specific refinements where needed, and keeps the final merged dataset traceable back to technology-level decisions. In addition, we implement a simple, technology-specific coding system for all records, where each study is assigned, an identifier consisting of a letter code for the technology and a running number reflecting its position after date-based sorting within that technology. This coding scheme is a critical element for managing files at scale, ensuring transparency in the processing workflow, and maintaining full traceability between raw exports, intermediate processing steps and the final integrated dataset. Specifically, we use the technology codes summarised in Table 1, combined with a running number (e.g. G-1, G-2, …, G-13540 for gasification) reflecting the sequence of records after date-based sorting within each technology.

**Table 1:** Technology-specific coding system used for record identifiers, applied consistently across all scripts, intermediate files and final dataset outputs.

Conversion technology	Code
Gasification	G
Pyrolysis	P
Fermentation	F
Anaerobic digestion	AnD
Aerobic digestion	AD
Hydrothermal liquefaction	HL
Torrefaction	To
Transesterification	Tr

### Finding relevant research

**Fig. 1:**
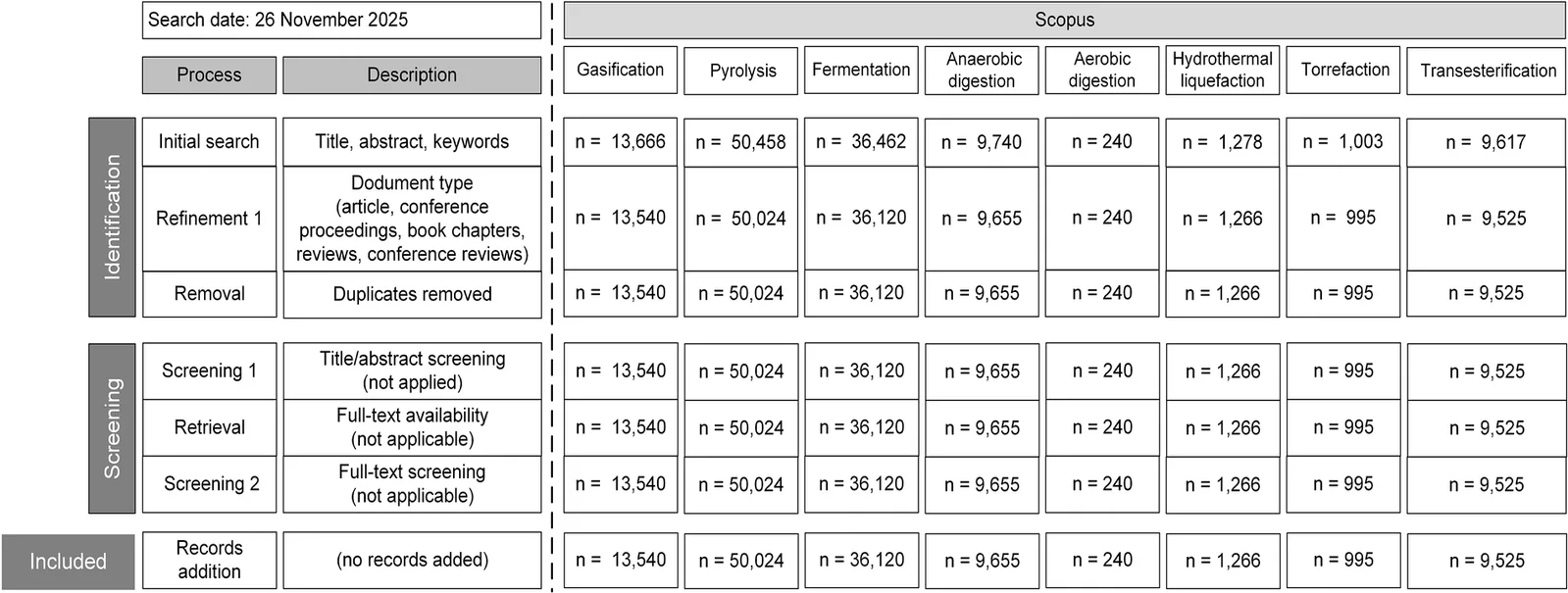
PRISMA-style overview of the literature identification and screening process for the eight major valorisation technologies. The diagram summarises Scopus searches (Title–Abstract–Keyword) conducted on 26 November 2025, subsequent refinement by document type, inspection of potential duplicates, and the absence of conventional title/abstract and full-text screening. Final counts correspond to all records kept for LLM-assisted data extraction for gasification, pyrolysis, fermentation, anaerobic digestion, aerobic digestion, hydrothermal liquefaction, torrefaction and transesterification.

The literature search is conducted using the Scopus database. Searches are run in the Title–Abstract–Keyword fields, covering all publications indexed up to the search cutoff shown in Fig. 1. No language restrictions are applied, as Scopus provides English titles and abstracts (or English translations) for the records used in this study. Following multiple pilot trials, the search strategy is refined through iterative testing of Boolean queries to balance recall and precision regarding the study aims. Separate searches are performed for each of the eight focal technologies. For each technology, the core query takes the following pattern:

TITLE-ABS-KEY (“<technology> of”)

for example, “gasification of” and “pyrolysis of”. This pattern is chosen because, in titles and abstracts, the phrase following “of” often names the processed material, which improves the extractability of feedstock mentions at scale. The trade-off is that some relevant records may describe the feedstock in alternative syntactic positions (e.g. preceding the technology term, embedded elsewhere in the sentence, or expressed via adjectival forms), and some papers may not state the feedstock explicitly in the title/abstract even when specified in the full text; such cases may therefore not be retrieved or may be non-informative for title–abstract extraction under this constrained query. The dataset should be interpreted as a high-precision, harmonised subset of the Scopus Title–Abstract–Keyword literature, designed to maximise consistency and accuracy in downstream LLM-assisted extraction and validation and to minimise the burden of manual curation.

After initial retrieval, records are restricted to relevant document types (articles, conference papers, book chapters, reviews and conference reviews). This refinement step reduces the number of records to 13,540 for gasification, 50,024 for pyrolysis, 36,120 for fermentation, 9,655 for anaerobic digestion, 240 for aerobic digestion, 1,266 for hydrothermal liquefaction, 995 for torrefaction and 9,525 for transesterification (Fig. 4). During preliminary checks we observed a small number of potential duplicate records within the Scopus export, typically cases where titles were identical, but abstracts differed, or where abstracts and DOIs were missing so records could not be reliably distinguished as true duplicates. To avoid erroneous exclusion of distinct studies, and because the possible duplicates are negligible relative to the overall scale of the dataset, we did not apply an explicit duplicate-removal step. Summed over all technologies, the final search corpus thus comprises 121,365 records.

Because the study relies exclusively on titles and abstracts for information extraction, access restrictions and paywalls do not affect data collection. In cases where Scopus labels a record as [No abstract available], the entry is kept, and downstream analysis is performed only on the title field. No conventional title/abstract or full text screening is carried out before data extraction. Instead, screening is integrated into the LLM-assisted extraction workflow: during processing, records that the model flags as clearly out of scope for the target technology (for example, non-biogenic feedstocks or non-conversion uses of the term) are assigned a “not applicable” label. This approach preserves the breadth of the initial search while managing the very large dataset in a scalable and reproducible manner.

To contextualise the effect of the intentionally constrained query structure, Table 2 contrasts the retrieval counts from technology-only searches with those obtained from the corresponding “[technology] of” searches within the same Scopus Title–Abstract–Keyword fields. Across the eight technologies, the constrained query yields 14–40% of the technology-mention corpus and 21% in aggregate (121,365 of 587,550 records). This reduction reflects the fact that, in many publications, the processed material is expressed in alternative syntactic positions (e.g. preceding the technology term, embedded elsewhere in the sentence, or via adjectival forms), is not stated explicitly in the title/abstract even when reported in the full text, or is not applicable. Importantly, the yield percentage quantifies only the effect of the query constraint on corpus size and should not be interpreted as recall or literature coverage. Instead, it operationalises a deliberate precision-oriented trade-off: by restricting retrieval to a construction that frequently makes the processed material explicit in the title/abstract, the resulting corpus is enriched in records with extractable feedstock mentions, which materially improves the accuracy and tractability of downstream LLM-assisted extraction and validation and substantially reduces the burden of manual curation. Despite the constraint, the kept set remains very large and diverse, providing a sufficiently rich basis for cross-technology comparisons of feedstock portfolios while preserving a transparent and reproducible linkage between retrieval logic and extractable evidence.

**Table 2:** Effect of the constrained “[technology] of” query relative to technology-only searches in Scopus.

	Retrieval counts for Scopus queries		
	n(“[technology]”)	n(“[technology] of”)	Constrained-query yield (%)
Gasification	59,176	13,540	23%
Pyrolysis	192,381	50,024	26%
Fermentation	249,690	36,120	14%
Anaerobic digestion	47,115	9,655	20%
Aerobic digestion	1,118	240	21%
Hydrothermal liquefaction	3,173	1,266	40%
Torrefaction	3,846	995	26%
Transesterification	31,051	9,525	31%
Total	587,550	121,365	21%

For each conversion technology, the table reports the number of records returned by a technology-only query (“[technology]”) and by the harmonised constrained query (“[technology] of”) when applied to the same Scopus Title–Abstract–Keyword fields. The percentage equals n(“[technology] of”) divided by n(“[technology]”) and quantifies the effect of the query constraint; it should not be interpreted as recall or literature coverage.

**Fig. 2:**
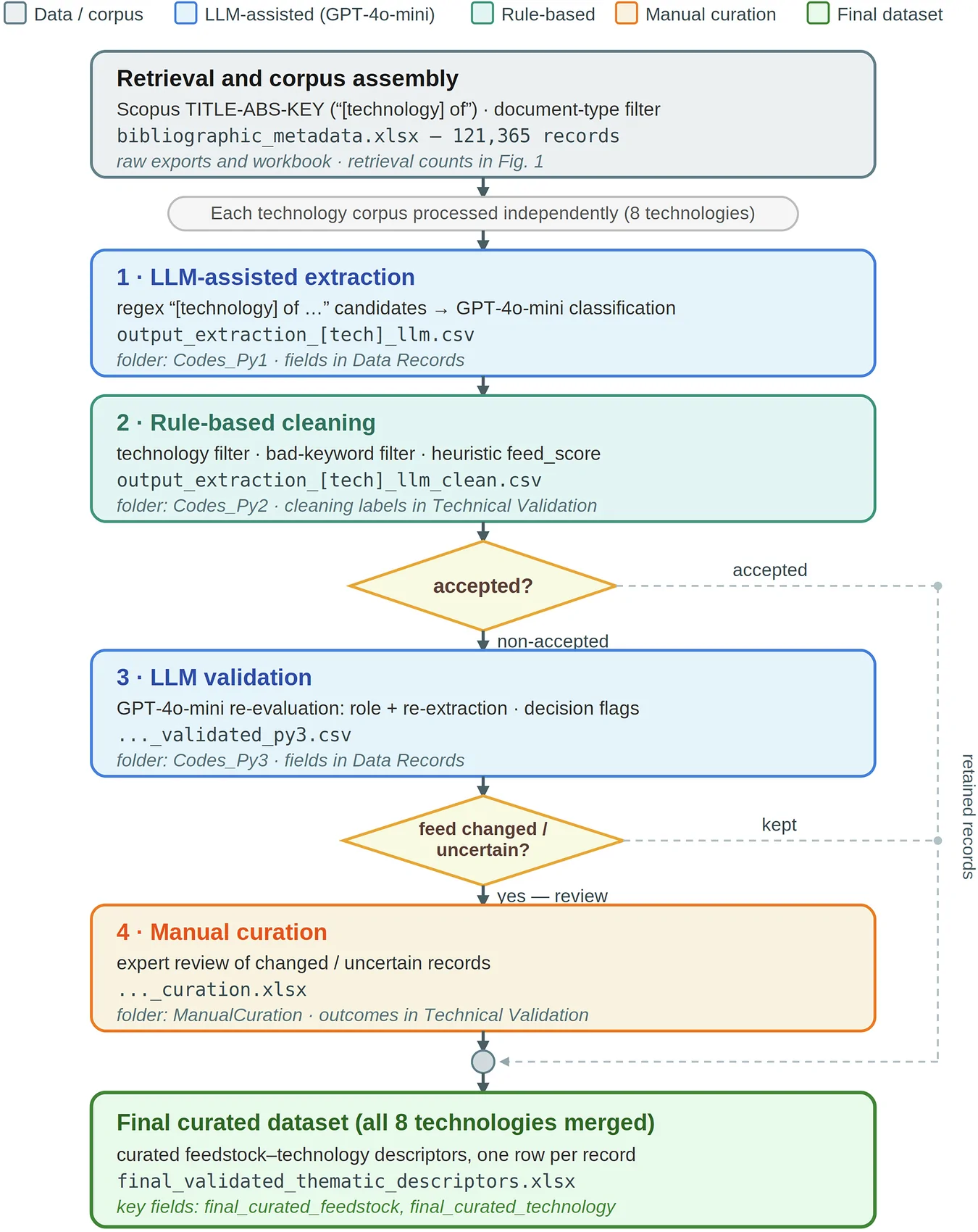
Workflow for dataset construction, extraction, validation and curation. The diagram summarises the end-to-end workflow from Scopus retrieval and corpus assembly to the final curated feedstock–technology dataset. Each technology corpus was processed independently through LLM-assisted extraction, rule-based cleaning, targeted LLM validation of non-accepted records and manual curation of changed or uncertain cases. Records accepted after rule-based cleaning and records kept after LLM validation were retained without further review and merged with manually curated records to form the final dataset. Colours distinguish data/corpus inputs, LLM-assisted steps, rule-based processing, manual curation and the final dataset.

To clarify how the retrieved records were converted into the final curated dataset, Fig. 2 summarises the complete data-processing workflow from Scopus retrieval to final dataset assembly. After corpus construction, each technology corpus was processed independently through LLM-assisted extraction, rule-based cleaning, targeted LLM validation and manual curation. The figure also shows the main repository files generated at each stage, thereby linking the methodological steps described below to the data and code structure provided in the repository.

### Data extraction

For each technology, we start from the corresponding Scopus Comma-separated values (CSV) export. The number of downloaded records for each technology is summarised in Fig. 4, where the combined corpus across all eight technologies comprises 121,365 records. During export, we select a standard set of metadata fields from Scopus (e.g. authors, year, source title, DOI, document type, language, authors keywords, publisher, title and abstract). Among the exported fields, assigned record identifier, article title and abstract are used as the primary textual inputs for the LLM-assisted extraction, while the remaining bibliometric fields are kept in the dataset for reference and potential downstream analyses but are not used directly in the extraction pipeline.

Data extraction is performed separately for each of the eight technologies using dedicated Python scripts that share the same core logic but differ in the value of the “base technology”. All scripts call the GPT-4o mini model via the ChatGPT API, with temperature set to zero and responses requested in structured JSON format to support reproducibility and downstream parsing. The extraction workflow proceeds in two main stages. First, we identify candidate feedstock phrases using regular expressions that search for patterns of the form “ < technology > of …” in titles and abstracts (for example, “gasification of…”, “pyrolysis of…”, “fermentation of…”). Candidate spans following “of” are cleaned to remove trailing clauses and stop words, while keeping multi-feed expressions (e.g. lists separated by commas). This pattern-based step provides an explicit, interpretable link between the named technology and nearby terms likely to denote feedstocks.

Second, the list of candidate phrases, together with the full title and abstract, is passed to the LLM. For each candidate, the model is prompted to decide whether it represents a true feedstock being processed by the given technology in that paper, or a non-feed term (e.g. products, intermediates, ash, slag). For candidates classified as valid feedstocks, the model returns: (i) a feed_verbatim string copied exactly from the text, (ii) a feed_final string representing the clearest short feedstock phrase still copied from the text, and (iii) a technology_phrase string that names the specific technology variant and contains the base technology term (e.g. “steam biomass gasification”, “fast pyrolysis of plastic waste”). Simple heuristic scoring, based on the presence of typical feedstock keywords and phrase length, is then used to select the most representative feedstock entry for each record. If no suitable feedstock is identified from the candidate-based step, a global fallback is applied: the title and abstract are sent to the model with a different prompt asking it to infer the main feedstock and technology phrase directly from the full context, still under the constraint that all returned phrases must be exact substrings of the original text. If the model can identify no plausible feedstock–technology pair, the record is assigned “not_applicable” for the feed and technology fields.

For each record, the final extraction output consists of the original metadata plus these additional fields: a base technology label (one of the eight technologies), the extracted technology_phrase, feed_verbatim, feed_final and an extraction_error field (see Data Record) that records any exceptions found during processing. Checkpointing is implemented so that intermediate results are periodically written to disk, ensuring robustness against interruptions. The same general pipeline is applied to all eight technologies, with only the base technology term and minor prompt wording always adapted, which guarantees methodological consistency across the full dataset. The extracted data then undergo a subsequent cleaning, validation and manual curation phase, as detailed in the Technical Validation section.

## Data Record

All data are available on DataverseNO^65^ in open access under a Creative Commons Attribution 4.0 International License (CC BY 4.0). The public release contains source identifiers, bibliographic metadata, extracted and curated descriptors, derived validation fields, code and documentation; Scopus-derived abstracts are not redistributed. Where an abstract column is retained in the deposited files, it is kept only for workflow/schema compatibility and the cells are left empty in the public release. The repository is structured to include processed and derived data, together with all Python scripts, source identifiers and auxiliary files required to document and reproduce the workflow where users have access to the source corpus. The repository is organised into one main data directory, three code directories, and a readme file. The overall folder structure is:

i. Review_DataExtraction_Validation_Curation_FinalDataset.zip
  - bibliographic_metadata.xlsx
  - DataExtraction/
  - DataCleaning/
  - DataValidation/
  - ManualCuration/
  - final_validated_thematic_descriptors.xlsx
ii. Codes_Py1_Automated_data_extraction_llm.zip
  [one subfolder per technology]
iii. Codes_Py2_Automated_extracted_data_cleaning.zip
  [one subfolder per technology]
iv. Codes_Py3_Automated_validation_llm.zip
  [one subfolder per technology]
v. ReadME.txt

The folders (Codes_py1-3) each contain subfolders named for the individual technologies, within which the relevant data, Python scripts, example input and output files, and brief manuals are stored. This structure reflects the technology-specific processing streams described in the Methods section and ensures a consistent, transparent linkage between code, intermediate outputs and the final data products. The subsequent subsections describe in detail the contents and internal structure of each archive.

### Review_ DataExtraction_Validation_Curation_FinalDataset/

This directory contains all primary data sources and derived datasets used in the study. It includes the bibliographic metadata exported from Scopus, the outputs of the LLM-assisted data extraction step, the intermediate datasets produced during cleaning, validation and manual curation, and the final validated thematic descriptors, which constitute the main data product of this work. The key components and a detailed description of their variables are provided in the following.

- bibliographic_metadata.xlsx – This workbook provides the consolidated set of Scopus records that form the bibliographic backbone of the study. It contains all publications retrieved for the eight technologies after applying basic document-type filters, with potential intra-database duplicates kept as discussed in the Methods section. Each record is assigned a unique record_id according to the coding system in Table 1, which serves as the primary key linking bibliographic information to all downstream extraction, validation and thematic descriptor tables. The workbook includes:
  a *cover* sheet with brief project, author, affiliation and date information (for documentation only; not intended for machine processing);
  a *search_log* sheet documenting, for each technology, the Scopus search strategy, including Boolean query strings, applied filters and other database refinements;
  one data sheet per technology, named using the pattern “technology_number-of-records” (e.g. *gasification_13540*, *pyrolysis_50024*), each containing the metadata fields described in Table 3.

**Table 3:** Bibliographic metadata fields included in bibliographic_metadata.xlsx.

Column name	Description
record_id	Unique study identifier assigned in this work.
article_title	Publication title retrieved from Scopus.
year	Publication year reported by Scopus.
source_title	Journal, proceeding, book series or source title.
doi	DOI, if available.
abstract	Abstract text was used internally for extraction and validation but is not redistributed in the public release.
author_keywords	Author-supplied keywords, if available.
publisher	Publisher listed by Scopus.
language	Document language reported by Scopus.
document_type	Scopus document type after filtering.
- DataExtraction/ – This directory contains one file per technology with the raw LLM extraction results produced by the script (*py1_data_extraction_[technology]_llm.py*). For each technology, the main output file follows the pattern *output_extraction_[technology]_llm.csv* and includes the extraction results, with columns defined and described in Table 4. The corresponding input files input_extraction_[technology].csv (containing record_id, article_title and abstract copied from the relevant sheet in *bibliographic_metadata.xlsx*) are stored together with the code in the Codes_Py1/ directory and are not duplicated in DataExtraction/.

**Table 4:** LLM-assisted extraction fields added to the DataExtraction outputs (output_extraction_[technology]_llm.csv).

Column name	Description
record_id	Unique study identifier.
article_title	Title used as extraction input.
abstract	Abstract field retained for workflow compatibility; abstract text was used internally during extraction but is left empty in the public release.
technology_base	Focal conversion technology used in the extraction prompt.
technology_phrase	Extracted technology phrase copied from the title or abstract; set to not_applicable if unresolved.
feed_verbatim	Feedstock phrase copied exactly from the title or abstract.
feed_final	Short feedstock descriptor copied from the source text; set to not_applicable if unresolved.
extraction_error	Processing, API or parsing error message; empty if none.
- DataCleaning/ – This directory contains one cleaned output file per technology generated by the automated cleaning and lightweight validation step (*py2_data_extraction_[technology]_llm_cleaning.py*). For each technology, the input file is the corresponding extraction output (output_extraction_[technology]_llm.csv from the DataExtraction stage). The main output is a cleaned dataset named *output_extraction_[technology]_llm_clean.csv* and is not duplicated in DataCleaning/. In this step, simple rule-based filters are applied to the LLM-derived fields to (i) reset obviously invalid technology or feedstock phrases to “not_applicable” (e.g. when they do not contain the expected technology term or include disqualifying keywords such as ash, slag or intermediates), and (ii) compute a heuristic feed_score and assign a categorical validation_status flag for each record. The cleaned file therefore has the same columns as output_extraction_[technology]_llm.csv, plus the two additional fields feed_score and validation_status; these newly added columns are defined and described in Table 5.

**Table 5:** Cleaning and lightweight validation fields added in the DataCleaning stage (output_extraction_[technology]_llm_clean.csv).

Column name	Description
feed_score	Heuristic confidence score assigned to the extracted feed_final phrase. The score increases when the phrase contains common feedstock anchors such as “waste”, “biomass”, “sludge”, “residue”, “MSW”, “plastic” or similar terms, and decreases when the phrase contains terms that usually indicate non-feed materials, products or process intermediates. The score is used only as a triage indicator for validation, not as a final measure of correctness.
validation_status	Rule-based cleaning label assigned after technology-term checks, bad-keyword filtering and feed-score evaluation. The label indicates whether a record is accepted directly or flagged for further validation, with categories such as accepted, suspect_low_confidence, suspect_missing_feed, rejected_non_[technology] and rejected_bad_keyword.
- DataValidation/ – This directory contains one validated output file per technology generated by the LLM-assisted validation script (*py3_[technology]_llm_cleaning_validation.py*). For each technology, the script takes as input the cleaned dataset *input_extraction_[technology]_llm_plus_cleaning.csv* and corresponds to the extraction output after the cleaning phase. It contains the columns from the extraction and cleaning stages, including record_id, article_title, the retained but empty abstract column, technology_base, technology_phrase, feed_verbatim, feed_final, technology_phrase_cleaning, feed_verbatim_cleaning, feed_final_cleaning, extraction_error, feed_score_cleaning and validation_status_cleaning. Abstract text was used internally for validation but is not redistributed in the public release. The validation script uses validation_status_cleaning to select only uncertain or problematic records for LLM-based re-evaluation, while records already marked as accepted in the cleaning step are typically kept without re-validation.
  The main output file is named *output_extraction_[technology]_llm_plus_cleaning_validated_py3.csv* and contains all input columns plus six additional phase-3 validation fields, defined in Table 6.

**Table 6:** Final LLM-based validation fields added to output_extraction_[technology]_llm_plus_cleaning_validated_py3.csv.

Column name	Description
[technology]_role_py3	Technology-role classification assigned during phase-3 LLM validation. The field indicates whether the focal technology is used as the primary conversion route, is only related to the study, or is not applicable for the target technology. Technology-specific values follow the form [technology]_primary, [technology]_related, not_[technology], or not_checked, depending on the validation outcome.
technology_phrase_validated_py3	Final validated technology phrase after phase-3 LLM validation. The phrase is copied from the title or abstract where possible and is set to not_applicable when no valid technology phrase is identified or when the record is classified as outside the target technology scope.
feed_final_validated_py3	Final validated feedstock phrase after phase-3 LLM validation, possibly updated relative to the original feed_final and optionally refined through abbreviation expansion when the abbreviation is explicitly defined in the title or abstract. The field is set to not_applicable when no clear feedstock is identified or when the record is classified as outside the target technology scope.
technology_phrase_decision_py3	Decision flag comparing technology_phrase_validated_py3 with the original technology_phrase. Values indicate whether the original technology phrase was kept, changed, or not checked during phase-3 validation.
feed_final_decision_py3	Decision flag comparing feed_final_validated_py3 with the original feed_final. Values indicate whether the original feedstock phrase was kept, changed, or not checked during phase-3 validation.
validation_note_py3	Short note documenting the reasoning for the phase-3 validation outcome, including any correction made by the LLM, reasons for classifying a record as not_[technology], or error messages if a validation call failed.
- ManualCuration/ – This folder contains one Excel workbook per technology, with filenames following the pattern *output_extraction_[technology]_llm_plus_cleaning_validated_py3_curation.xlsx*. Each file is generated from the corresponding validation output (*output_extraction_[technology]_llm_plus_cleaning_validated_py3.csv*) and includes four additional columns used in the manual curation step (Table 7). In this stage, records flagged as changed or uncertain by the automated validation (for example, those with feed_final_decision_py3 = “changed”) are reviewed manually. When the curator accepts the LLM-validated values, the curation decision fields are left empty, and the curated fields remain identical to the validated entries. When the curator does not agree with the automated validation, the decision fields are populated with an appropriate label, and the curated fields are overwritten with the corrected feedstock or technology phrase.

**Table 7:** Manual curation fields added in the Manual Curation stage.

Column name	Description
decision_curation_feed_py3	Decision flag for the feedstock field at the manual curation stage. This field is left blank when the curator accepts ‘feed_final_validated_py3‘ as correct and is filled in (with any non-empty marker) when the curator does not agree with the validated feed and provides a different curated value.
curated_feed_py3	Final curated feedstock phrase. By default, initialised to the value of ‘feed_final_validated_py3‘; updated only for records where the curator corrects or refines the LLM-validated feedstock.
decision_curation_technology_py3	Decision flag for the feedstock field at the manual curation stage similar to decision_curation_feed_py3.
curated_technology_py3	Final curated technology phrase. By default, identical to ‘technology_phrase_validated_py3‘; modified only for records where the technology phrase is manually corrected during curation.
- final_validated_thematic_descriptors.xlsx – This workbook contains the fully curated output intended for reuse in subsequent analyses. It represents the final, reconciled dataset after automated extraction, cleaning, LLM-based validation and manual curation. The file includes a cover sheet with basic project and documentation information, and one data sheet per technology, named using the full technology name. For consistency across the repository, each technology sheet keeps all bibliographic and metadata fields from *bibliographic_metadata.xlsx* and adds two final curation fields, final_curated_feedstock and final_curated_technology. These two fields are the user-facing counterparts of curated_feed_py3 and curated_technology_py3 generated by the validation–curation pipeline and provide the final, preferred feedstock and technology descriptors for each record.

### Codes_Py1_Automated_data_extraction_llm/

This directory contains the scripts and auxiliary files used for the LLM-assisted data extraction step. It is organised into one subfolder per technology, and each technology-specific subfolder contains:

1. py1_data_extraction_[technology]_llm.py – The Python script implementing the phase 1 extraction workflow for the given technology (as described in the Methods – Data extraction section).
2. py1_data_extraction_[technology]_llm.txt – A plain-text version of the script or its core prompt and configuration, provided for quick inspection and version control without opening the Python file.
3. input_extraction_[technology].csv – The input file to the extraction script, containing record_id, article_title and an abstract column. Abstract text was used internally during extraction, but in the public release the abstract column is retained only for workflow compatibility and left empty.
4. output_extraction_[technology]_llm.csv – The main output of the extraction step for that technology, with columns defined in Table 4.
5. user_manual_py1_data_extraction_[technology]_llm.pdf – Detailed documentation outlining installation requirements, input/output format, workflow configuration, and practical examples.

### Codes_Py2_Automated_extracted_data_cleaning/

This directory contains the scripts and supporting files used for the automated cleaning and lightweight validation of the extraction results from Codes_Py1. As before, it is organised into one subfolder per technology, and each technology-specific subfolder includes:

1. py2_data_extraction_[technology]_llm_cleaning.py – The Python script implementing the phase 2 rule-based cleaning and scoring of the LLM-extracted fields, as described in the DataCleaning stage.
2. py2_data_extraction_[technology]_llm_cleaning.txt – A plain-text version of the script or its core prompt and configuration, provided for quick inspection and version control without opening the Python file.
3. output_extraction_[technology]_llm.csv – The direct output from the phase 1 extraction script Codes_Py1, used here as the input file for the cleaning step.
4. output_extraction_[technology]_llm_clean.csv – The cleaned output produced by the phase 2 script, containing the same columns as input plus the additional fields defined in Table 5.
5. user_manual_py2_data_extraction_[technology]_llm_cleaning.pdf – Detailed documentation outlining installation requirements, input/output format, workflow configuration, and practical examples.

### Codes_Py3_Automated_validation_llm/

This directory contains the scripts and related files used for the LLM-based validation of the extracted results, building on the outputs of the data extraction and cleaning step. As with the previous code folders, it is organised into one subfolder per technology, and each technology-specific subfolder contains:

1. py3_[technology]_llm_cleaning_validation.py – The Python script implementing the final LLM-assisted validation workflow for the given technology, as described in the *DataValidation* subsection.
2. py3_[technology]_llm_cleaning_validation.txt – A plain-text version of the script or its core prompt and configuration, provided for quick inspection and version control without opening the Python file.
3. input_extraction_[technology]_llm_plus_cleaning.csv – The input file to the validation script, containing the combined extraction and cleaning outputs for that technology. Its structure and columns are described in section e. *DataValidation*.
4. output_extraction_[technology]_llm_clean.csv – The validated output produced by the phase 3 script, which keeps all input columns and adds the validation-specific fields defined in Table 6.
5. user_manual_py3_[technology]_llm_cleaning_validation.pdf

## Data Overview

**Fig. 3:**
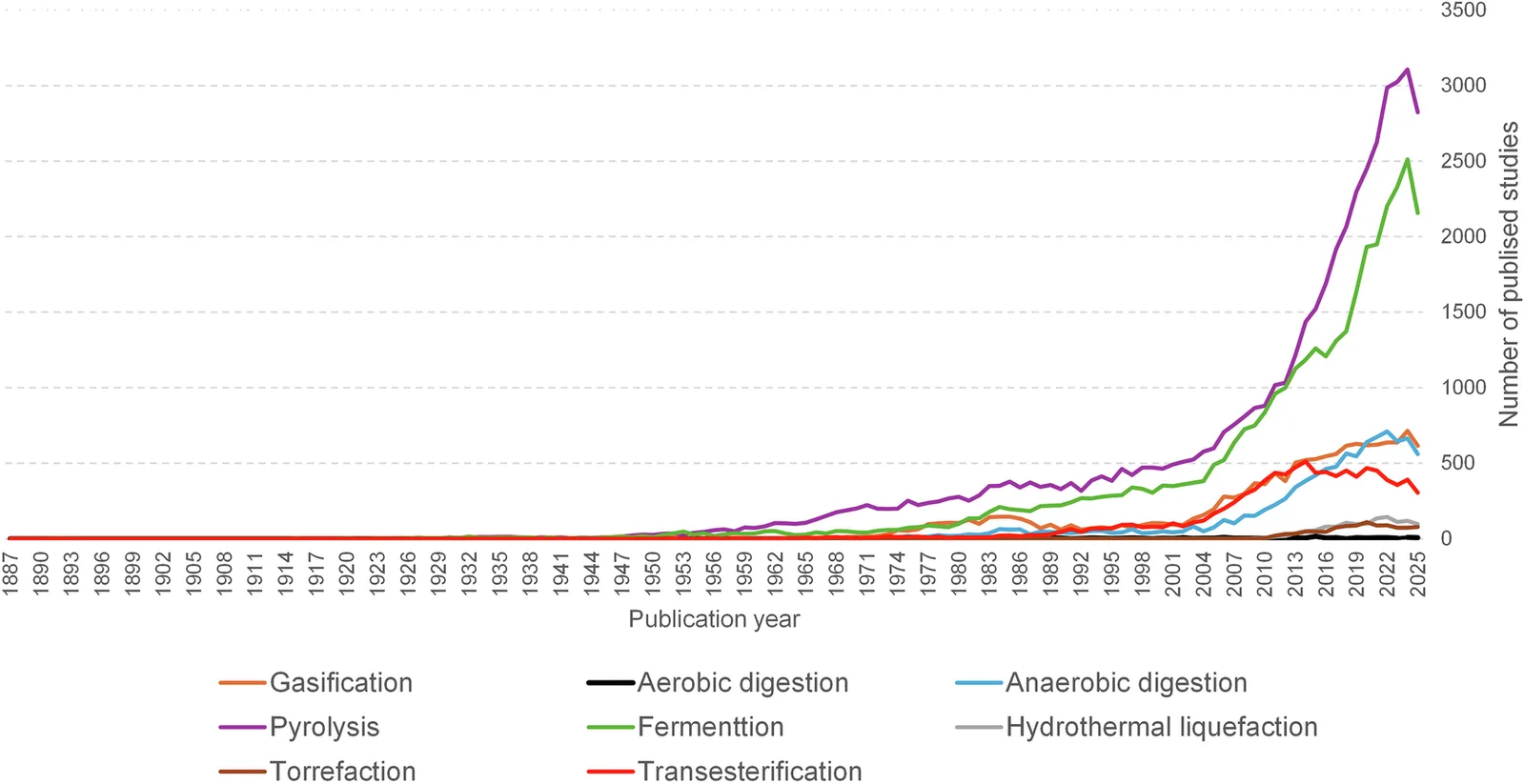
Temporal evolution of Scopus-indexed publications retrieved with the technology-specific search strings for the eight biomass and waste conversion technologies (gasification, pyrolysis, fermentation, anaerobic digestion, aerobic digestion, hydrothermal liquefaction, torrefaction and transesterification). The curves show annual publication counts from the first retrieved record for each technology up to 2025; the apparent decline in 2025 reflects the partial coverage of that year at the time of the search. A few early-indexed 2026 records were present in Scopus but are not plotted for clarity (gasification: 46; pyrolysis: 184; fermentation: 44; anaerobic digestion: 17; hydrothermal liquefaction: 13; transesterification: 14; torrefaction: 4; aerobic digestion: 0).

The assembled dataset spans more than a century of research activity and captures the temporal evolution of biomass and waste conversion technologies (Fig. 3). The earliest records are observed for fermentation (first Scopus-indexed paper in 1887), reflecting its long-standing role in industrial biotechnology. Pyrolysis and gasification emerge shortly thereafter, with first records in 1930 and 1931, respectively, followed by anaerobic digestion in 1941 and transesterification in 1945. In contrast, aerobic digestion (1973), torrefaction (2006) and hydrothermal liquefaction (2008) appear much later in the literature, consistent with their development as more recent or specialised options for waste and residue upgrading. Across all technologies, publication volumes remain modest until the 1970s–1980s, after which gradual growth is observed, followed by a pronounced acceleration from the early 2000s onwards, coinciding with the rise of the bioeconomy, climate policy targets and increasing interest in waste valorisation. The apparent drop in 2025 simply reflects the search having been conducted late in the year and therefore captures only a partial publication year.

**Fig. 4:**
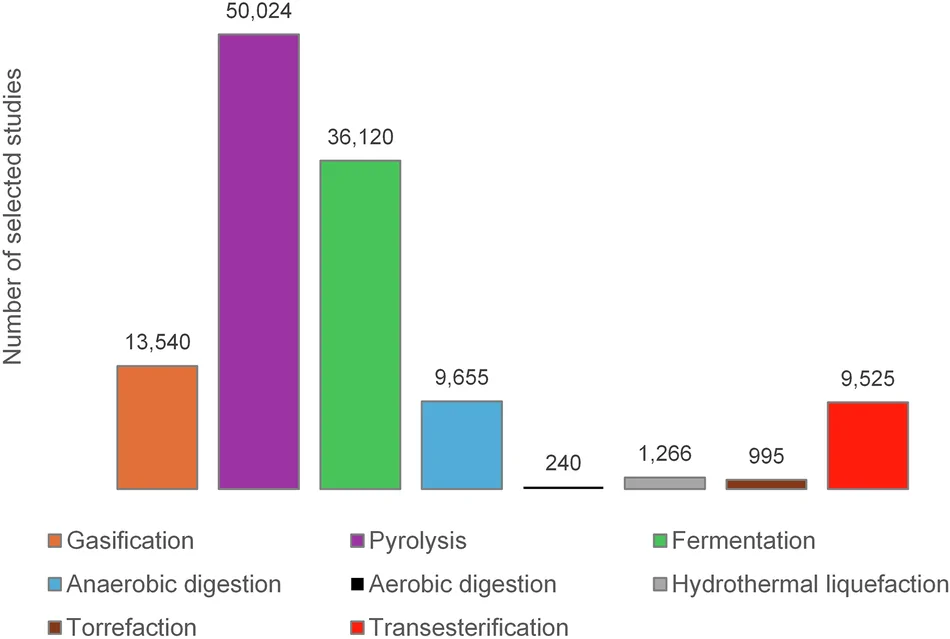
Number of publications retrieved for each conversion technology using the harmonised “technology of …” search strings in Scopus (total n = 121,365). The counts represent the LLM-ready subset of the literature used for feedstock–technology mapping in this study, rather than the full global corpus for each technology.

Regarding scale, the final screened corpus derived from our search strategy comprises 121,365 records across the eight technologies (Fig. 4). These counts do not represent the complete global literature for each technology; rather, they reflect the subset of publications retrieved with our deliberately constrained “[technology] of” search strings, which were chosen to maximise the likelihood that a concrete feedstock is explicitly mentioned and thus extractable. Within this LLM-ready corpus, pyrolysis and fermentation dominate numerically, with 50,024 and 36,120 records, respectively, highlighting their central role in both thermal and biochemical conversion research. Gasification accounts for 13,540 records, while anaerobic digestion and transesterification contribute 9,655 and 9,525 studies, respectively. Aerobic digestion is represented by 240 records, reflecting its narrower, more application-specific reporting under the chosen query structure, whereas hydrothermal liquefaction and torrefaction contribute 1,266 and 995 studies. Although these numbers undercount the total literature in each field, they delineate a very large and systematically constructed subset that is particularly well suited for comparative analysis of feedstock–technology combinations and for characterising the practical decision space across conversion pathways.

To demonstrate feedstock-level utility beyond bibliographic screening, we derived summary metrics from the final curated feedstock descriptors and the harmonised L1 feedstock-labelling outputs deposited in a companion DataverseNO record^66^. This companion record was developed as a continuation of the present dataset: it builds on the curated feedstock–technology descriptors reported here by splitting multi-material feedstock phrases into single feedstock items, where “splitting” means separating combined phrases such as “biomass and plastic waste” into individual feedstock entries, and by assigning harmonised labels for renewability and material status. The source feedstock descriptors therefore come from the present dataset, whereas the post-splitting item-level counts, valid/not-applicable status and renewability profiles reported in Table 8 are derived from the companion feedstock-labelling record. The table is therefore item-level, based on 143,353 post-splitting feedstock items, and should be distinguished from the 121,365 bibliographic records in the core dataset. For the core eight-technology dataset, 124,855 items were classified as valid feedstock items and 18,498 as not applicable.

**Table 8:** Feedstock-level summary metrics derived from the final curated feedstock descriptors and harmonised L1 labels.

Technology	Total feedstock items	Valid feedstock items	Not applicable, n (%)	Renewable (%)	Non-renewable (%)	Mixed/unknown (%)
G	15,922	14,664	1,258 (7.9%)	46.9	40.5	12.6
P	61,006	50,074	10,932 (17.9%)	39.5	52	8.5
F	41,610	36,536	5,074 (12.2%)	90.6	2.8	6.6
AnD	10,993	10,810	183 (1.7%)	60.5	1.5	38
AD	260	249	11 (4.2%)	23.3	0	76.7
HL	1,439	1,436	3 (0.2%)	85.5	2.4	12
To	1,218	1,215	3 (0.2%)	88.8	3.2	8
Tr	10,905	9,871	1,034 (9.5%)	72.8	25	2.2
Overall	143,353	124,855	18,498 (12.9%)	60.7	28.6	10.6

Total feedstock items refer to post-splitting feedstock items generated from the curated feedstock descriptors, where post-splitting means that combined feedstock phrases were separated into individual feedstock entries. Valid feedstock items are entries retained as usable feedstocks after labelling and validation. The source feedstock descriptors originate from the present dataset, whereas the post-splitting item-level counts, valid/not-applicable status and renewability-profile columns are derived from the harmonised feedstock-labelling outputs deposited in a companion DataverseNO record^66^. The not-applicable percentage is calculated relative to total feedstock items, whereas renewability percentages are calculated relative to valid feedstock items.

Table 8 reports the feedstock-level distribution across technologies. Pyrolysis and fermentation contain the largest valid feedstock-item sets, while smaller corpora such as aerobic digestion, torrefaction and hydrothermal liquefaction still show distinct feedstock profiles. Renewability profiles differ systematically across technologies (χ²(14, N = 124,855) = 42,615, p < 0.001; Cramér’s V = 0.41). Substantively, this means that the feedstocks reported for different technologies differ in renewability: some technologies are mostly linked to renewable feedstocks, whereas others show much higher shares of non-renewable or mixed/unknown items. Because of the large item-level sample size, the p-value should be interpreted cautiously; the effect size indicates a moderate association. This confirms that the dataset contains statistically differentiated feedstock–technology patterns, rather than only technology-level publication counts. Fermentation, torrefaction and hydrothermal liquefaction are predominantly renewable (85–91%); pyrolysis has the largest non-renewable share (52%); transesterification is mostly renewable (73%) but has a substantial non-renewable component (25%); anaerobic digestion is mainly renewable (61%) but has a high mixed/unknown share (38%); and aerobic digestion is dominated by mixed/unknown entries (77%), reflecting its small and often under-specified item set. These metrics show that the dataset can support feedstock-portfolio comparison, targeted retrieval and evidence-gap mapping, rather than serving only as an initial screening output.

## Technical Validation

This section describes the validation approach used to ensure traceability and reliability at scale. It is structured in four parts: (i) the overall validation strategy, which motivates the layered design; (ii) the rule-based cleaning of extraction outputs, which applies deterministic filters and confidence scoring to all records; (iii) the LLM-based validation on the initial extraction, which performs targeted re-evaluation and supports subsequent manual curation of flagged cases; and (iv) summary statistics that quantify the outcomes of the automated cleaning and validation steps across technologies.

### Overall validation strategy

Before implementing any automated checks, we defined a validation strategy tailored to the scale and purpose of the dataset. The key design choice was to couple a very large literature corpus with an extraction logic that is simple and interpretable. Based on lessons from our previous large-scale data descriptors (submitted/under review^61–64^), we recognised that the search string itself strongly affects both extraction accuracy and the difficulty of downstream validation specifically in LLM-assisted applications. We therefore adopted technology-specific queries of the form “[technology] of” (for example, “gasification of”, “pyrolysis of”), which simultaneously: (i) yield large and diverse corpora across all eight technologies, and (ii) create a regular expression (regex) syntactic pattern in titles and abstracts that helps to localise feedstock phrases for both regex-based pre-processing and LLM interpretation.

Validation was then organised in three layers:

1. Rule-based cleaning: lightweight, fully deterministic filters and scores applied to every extracted record. This step is not used as ground truth, but to flag cases where the LLM extraction is likely unreliable and to provide an additional option that the validation model can compare against.
2. LLM-based validation: a second, independent pass with GPT-4o-mini that re-reads titles and abstracts and re-assesses the main technology phrase and feedstock, guided (but not constrained) by the cleaning status.
3. Manual curation: targeted expert review of records where the automated validation indicates changes or persistent uncertainty.

Together, these layers are designed to maximise scalability while keeping all corrections transparent and traceable.

### Rule-based cleaning of extraction outputs

The cleaning scripts implement a lightweight, rule-based “sanity check” of the LLM extraction for each technology. They all follow the same general structure as the example given for pyrolysis:

- For each record, the script reads the extracted technology_phrase, feed_final and feed_verbatim.
- A hard technology filter first checks whether the technology phrase follows the target technology. For example, in the pyrolysis case the phrase must contain “pyrolysis”, “pyrolytic”, “pyrolyzer/pyrolyser”, or other clear variants. If this condition is not met, both technology and feed fields are reset to not_applicable and the record is labelled rejected_non_[technology].
- A second hard filter on “bad” keywords screens out phrases that clearly refer to non-feed materials or non-primary outputs (e.g. “slag”, “fly ash”, “char”, “catalyst”, “tar”, “syngas”, “bio-oil”, “reaction intermediates”). If such terms appear in either the technology or feed field, the record is labelled rejected_bad_keyword and the corresponding fields are nulled.
- For records that pass these hard filters, a heuristic feed score is computed. The scoring function rewards the presence of canonical feedstock terms (e.g. “waste”, “biomass”, “sludge”, “residue”, “municipal solid waste”, “plastic”, “coal”) and penalises obviously unsuitable terms. A small bonus is added for longer, more specific phrases, capped to avoid favouring excessively verbose strings.
- Based on this score and the existence of a non-empty feed phrase, each record is assigned a validation_status:
  i. accepted – technology phrase is consistent and feed score is above the threshold;
  ii. suspect_missing_feed – technology is valid, but the feed is missing or not_applicable;
  iii. suspect_low_confidence – technology is valid, but the feed score is below the threshold;
  iv. rejected_non_[technology] or rejected_bad_keyword – as defined above.

The cleaning step adds two new columns to the extraction output: feed_score and validation_status. Importantly, cleaning phase is not treated as a final classifier. Its role is to (i) normalise obvious inconsistencies, (ii) pre-label records by confidence, and (iii) determine which rows are sent to the more expensive LLM-based validation.

### LLM-assisted validation and final manual curation on initial extraction

In the next phase, we perform an independent LLM-based validation that operates directly on the original titles and abstracts. The cleaning results are used only to *select* records for validation and to prioritise effort; they do not constrain what the validator can conclude. For each technology, validation step proceeds as follows (illustrated here with the pyrolysis implementation, but the same logic is adapted for the other technologies):

1. Record selection.
  Records with validation_status = accepted are generally trusted and *not* re-analysed by the LLM; they are carried forward with a role flag set to “primary” for the corresponding technology.
  Records with validation_status in an “uncertain” set (e.g. suspect_low_confidence, suspect_missing_feed, rejected_bad_keyword, rejected_non_[technology]) are passed to GPT-4o-mini for detailed validation.
2. Role classification and re-extraction – For each selected record, the model receives the title, abstract and the *initial* extraction (technology_phrase, feed_final). A structured prompt then asks the model to:
  - classify the role of the technology in the paper (e.g. pyrolysis_primary, pyrolysis_related, or not_pyrolysis; analogous labels are used for the other technologies);
  - identify its best estimate of the technology phrase and feed phrase, subject to strict constraints:
    both phrases must be exact substrings of the title or abstract, with the single allowance that abbreviations in parentheses may be dropped. For example, if the text contains “plastic waste (PW)”, the feed phrase may be given as “plastic waste”;
    the technology phrase must contain the technology keyword (e.g. “pyrolysis”);
    no expansion of undefined abbreviations is allowed;
    in certain domain-specific edge cases (e.g. pure catalyst precursor/MOF pyrolysis, or acetic-acid steam reforming for gasification) the model is instructed to set the feed to not_applicable and/or classify the record as not_[technology] for our mapping purposes.
3. Post-processing and decision flags – The script compares the LLM-validated phrases with the original extraction and sets:
  a final role flag (e.g. pyrolysis_role_py3),
  technology_phrase_validated_py3 and feed_final_validated_py3,
  decision indicators (technology_phrase_decision_py3, feed_final_decision_py3) recording whether each field was kept, changed, or not_checked in phase 3, and
  a short free-text validation_note_py3 summarising the rationale (e.g. “initial feed was ash; changed to municipal solid waste” or “feed set to not_applicable – MOF precursor only”).
  Deterministic post-processing also expands defined abbreviations in feed phrases. For example, if the title and abstract contain “plastic waste (PW)”, an extracted feed phrase of “PW” is replaced by “plastic waste”. A few additional rule-based corrections are applied (such as downgrading precursor-only feeds to not_applicable even when the LLM proposes them).
4. Manual curation layer – We primarily focus on the feedstock field, i.e. records where feed_final_decision_py3 = “changed”. These entries are reviewed manually. When the curator agrees with the LLM-validated value, the curation decision fields are left empty, and the curated fields simply mirror feed_final_validated_py3. When the curator disagrees, the decision fields are populated with a proper label (in this dataset, “updated”), and the curated feedstock (and, where relevant, technology) phrase overwrites the validated value, making sure every departure from the automated correction is explicitly documented.

This combination of rule-based cleaning, conservative LLM validation and explicit decision flags provides a transparent audit trail from the raw extraction to the final curated descriptors and lets users filter the dataset by confidence level or to re-interpret specific subsets if alternative assumptions are desired.

### Summary statistics for automated cleaning and validation

Table 9 summarises the distribution of cleaning labels produced in phase 2 for each technology. Across all technologies, records labelled accepted are carried forward without further automated checks, whereas all other categories (suspect_low_confidence, rejected_non_[technology], suspect_missing_feed, rejected_bad_keyword) are systematically passed to the phase 3 LLM-based validation. The suspect_low_confidence class is dominated by records where the extracted feedstock is essentially correct but expressed in a short or abbreviated form (for example, acronym-only feeds or minor wording variants); these are therefore typically subject to minor changes rather than major corrections in the validation and curation stages.

**Table 9:** Distribution of rule-based cleaning labels for each conversion technology (where technology codes are defined in Table 1).

Cleaning labels	G	P	F	AnD	AD	HL	To	Tr
accepted	63%	39%	29%	78%	88%	73%	73%	43%
suspect_low_confidence	15%	43%	43%	14%	8%	25%	23%	43%
rejected_non_[technology]	10%	11%	18%	3%	3%	0%	1%	8%
suspect_missing_feed	0%	2%	1%	0%	0%	0%	0%	0%
rejected_bad_keyword	12%	5%	9%	4%	1%	2%	3%	7%

Values show the percentage of records assigned to each label after cleaning. Records labelled accepted are kept as-is, while all other labels are forwarded to LLM-based validation. The suspect_low_confidence class mainly captures entries where the feedstock has been correctly detected but is expressed with abbreviations or very short phrases and typically requires only minor normalisation rather than substantive correction.

For most technologies (gasification, anaerobic/aerobic digestion, hydrothermal liquefaction and torrefaction), between roughly two-thirds and almost ninety per cent of records are immediately labelled as accepted, suggesting that the initial LLM extraction already provides a plausible technology phrase and feedstock. Pyrolysis and transesterification stand out with a markedly lower share of accepted records (around 40%) and a correspondingly higher fraction flagged as suspect_low_confidence, reflecting the more heterogeneous terminology and frequent mention of non-primary materials (e.g. chars, oils, intermediates) in these literatures. Fermentation shows the lowest accepted fraction in the rule-based cleaning stage (29%) through a combination of two effects. Like pyrolysis and transesterification, it has a high suspect_low_confidence share (43%), because fermentation feedstocks are often reported as short substrate names, sugars, or carbohydrate-rich commodity inputs that are valid substrates but lack the canonical feed anchors (e.g. “waste”, “biomass”, “sludge”, “residue”) rewarded by the heuristic feed_score. In addition, and unlike the other technologies, fermentation has the highest rejected_non_[technology] share (18%) together with an elevated rejected_bad_keyword share (9%). This reflects the broad use of the term “fermentation” across microbiology, food science, gut and digestive-system studies, pharmaceutical biotechnology and general biochemical laboratory studies, where it may appear without a clear waste- or biomass-to-X conversion role, or without an extractable feedstock–technology pair in the title or abstract. The lower accepted fraction for fermentation therefore reflects both the conservative cleaning rules and the generic, cross-domain usage of the term, and indicates that the cleaning step is screening out off-scope records rather than a failure of the extraction workflow.

Across all technologies, the proportions of *rejected_non_[technology]* and *rejected_bad_keyword* remain modest, suggesting that the “[technology] of …” search strategy rarely retrieves papers that are entirely outside scope, but is still sufficiently restrictive to filter obvious misclassifications. The *suspect_missing_feed* category is essentially negligible, which confirms that the chosen search pattern successfully exposes feedstock expressions in titles and abstracts.

Table 10 summarises how Phase 3 validation and the subsequent manual curation affected the feedstock and technology descriptors for each conversion route. For each technology, all percentages are expressed as a share of the total number of records for that technology. The column “checked” reports the fraction of records that were forwarded from rule-based cleaning to LLM validation, corresponding to all non-accepted cases from Table 9. “Kept” and “changed” partition this same fraction into records for which the original extraction was confirmed by the LLM or modified by the LLM, respectively, such that: checked ≈ kept + changed. The “updated” column shows the proportion of all records for which the curator overruled the LLM-validated value during manual curation.

**Table 10:** LLM-based validation and manual curation outcomes for feedstock and technology descriptors.

Technologies	Feedstock labels				Technology labels		
	Validation			Manual Curation	Validation		
	checked	kept	changed	updated	checked	kept	changed
Gasification	37%	30%	7%	0.5%	37%	28%	9%
Pyrolysis	61%	55%	6%	0.4%	61%	37%	24%
Fermentation	71%	61%	9%	3%	71%	43%	27%
Anaerobic Digestion	22%	18%	4%	0.2%	22%	17%	4%
Aerobic digestion	12%	11%	1%	0.0%	12%	10%	2%
Hydrothermal Liquefaction	27%	24%	3%	0.5%	27%	11%	17%
Torrefaction	27%	24%	3%	0.3%	27%	21%	6%
Transesterification	57%	51%	7%	0.6%	57%	39%	18%

For each technology, all percentages are calculated relative to the total number of records for that technology. “Checked” denotes the share of records sent from rule-based cleaning to LLM validation (all non-accepted cases). “Kept” and “changed” split this same share into records where the original extraction was kept or modified by the LLM. “Updated” indicates the fraction of all records for which manual curation overruled the LLM-validated feedstock or technology phrase.

For feedstocks, only about 3–7% of all records per technology are changed by the LLM, and fewer than 1% are subsequently updated by manual curation, suggesting that most extracted feeds are already robust. Technology phrases show somewhat higher change rates (up to about one quarter for pyrolysis and transesterification), reflecting more heterogeneous terminology, yet manual overrides remain rare. Overall, the combination of rule-based cleaning, LLM validation and manual curation results in a low fraction of modified feedstock descriptors (1–9% per technology), with manual overrides generally affecting fewer than 1% of records, except for fermentation where 3% of records were manually updated. This suggests that the initial LLM extraction is generally robust, while the validation pipeline effectively captures and corrects problematic cases.

## Usage Notes

The dataset can support several forms of reuse, including but not limited to the examples outlined below. First, because the search logic, code, curated fields, audit outputs and documentation are made available, the dataset can be reused not only as a data product but also as a scalable workflow for constructing similar evidence maps in other technology domains. Researchers can adapt the same approach to extract, clean, validate and curate feedstock–technology relationships, extend the evidence base with new records, or develop comparable datasets for other waste-to-X, bioeconomy and circular-economy applications.

Second, evidence-synthesis and systematic-review researchers can use the curated feedstock and technology fields, together with DOI, title, and bibliographic metadata, to retrieve studies and build evidence maps for specific feedstock–technology pairs, such as sewage sludge–hydrothermal liquefaction, plastic waste–pyrolysis or lignocellulosic residues–gasification. Third, researchers conducting LCA, TEA, and MCDA can use the dataset as a structured starting point for defining feedstock–technology alternatives before adding context-specific performance, cost, emissions or availability data.

Fourth, circular-economy and sustainability researchers can use the feedstock descriptors, together with companion harmonised labels where relevant, to compare feedstock portfolios across technologies, including renewability, material status, hazard relevance and circularity-interest profiles. Fifth, data-science, natural-language-processing and knowledge-graph researchers can reuse the curated feedstock–technology pairs and bibliographic metadata as a labelled corpus for feedstock extraction, classification, terminology structuring and knowledge-graph construction. The harmonised dictionaries and extended label outputs are available as companion resources, while the present dataset provides the core curated feedstock–technology evidence layer.

Sixth, industry users and technology developers can use the dataset for early-stage technology scouting by identifying reported feedstocks, feedstock diversity and under-explored feedstock–technology combinations. Seventh, policy, funding and roadmap analysts can use the dataset to identify where evidence is concentrated or sparse across feedstock–technology combinations, supporting research-priority setting and targeted evidence retrieval.

The dataset should be interpreted as a structured evidence resource rather than a direct measure of real-world feedstock availability, market scale, technology readiness or industrial deployment. It is most useful for sustainable-transition and resource-allocation studies when combined with regional or international resource-availability data, process-performance data and context-specific sustainability assessment.

The dataset has already supported companion reuse outputs, including harmonised feedstock-labelling records, derived summary metrics, label dictionaries, audit files and case-study calculations. These companion records demonstrate how the dataset can be extended from bibliographic evidence mapping to feedstock-portfolio comparison and circularity-oriented screening^66–68^.

## Data Availability

All supplementary materials including data generated or analysed during this study are deposited on DataverseNO in the following URL: https://doi.org/10.18710/JM6U7B. The public release contains the curated feedstock–technology descriptors, source identifiers, bibliographic metadata, derived validation fields, code and documentation. Scopus-derived abstracts are not redistributed; users with appropriate database access can reconstruct the input corpus using the documented search strings, search date and source identifiers.

## Code availability

All custom scripts used for LLM-assisted data extraction from Scopus metadata (Codes_Py1), rule-based cleaning and heuristic validation (Codes_Py2), and automated LLM-assisted validation (Codes_Py3) are openly available in the DataverseNO repository associated with this publication (https://doi.org/10.18710/JM6U7B). The folder structure and file list are described in the Data Records section. No parts of the analysis rely on proprietary or closed‐source code. The scripts document the full extraction and validation workflow and require title and abstract fields as inputs; however, the abstract text itself is not redistributed in the public deposit because it belongs to the source corpus.
